# Growth and Crystallization of SiO_2_/GeO_2_ Thin Films on Si(100) Substrates

**DOI:** 10.3390/nano11071654

**Published:** 2021-06-23

**Authors:** Jordi Antoja-Lleonart, Václav Ocelík, Silang Zhou, Kit de Hond, Gertjan Koster, Guus Rijnders, Beatriz Noheda

**Affiliations:** 1 Nanostructures of Functional Oxides, Zernike Institute for Advanced Materials, University of Groningen, 9747 AG Groningen, The Netherlands; j.antoja.lleonart@rug.nl (J.A.-L.); v.ocelik@rug.nl (V.O.); s.zhou@rug.nl (S.Z.); 2 MESA+ Institute for Nanotechnology, University of Twente, P.O. Box 217, 7522 NH Enschede, The Netherlands; c.a.j.dehond@utwente.nl (K.d.H.); G.Koster@utwente.nl (G.K.); a.j.h.m.rijnders@utwente.nl (G.R.)

**Keywords:** quartz, silica thin films, pulsed laser deposition, devitrifying agent, crystallization, electron back-scatter diffraction

## Abstract

The growth of α-quartz-based piezoelectric thin films opens the door to higher-frequency electromechanical devices than those available through top-down approaches. We report on the growth of SiO${}_{2}$/GeO${}_{2}$ thin films by pulsed laser deposition and their subsequent crystallization. By introducing a devitrifying agent uniformly within the film, we are able to obtain the α-quartz phase in the form of platelets with lateral sizes above 100 μm at accessible temperatures. Films containing different amounts of devitrifying agent are investigated, and their crystallinity is ascertained with X-ray diffraction and electron back-scatter diffraction. Our work highlights the difficulty in crystallization when competing phases arise that have markedly different crystalline orientation.

## 1. Introduction

α-Quartz is a well-known piezoelectric phase of SiO${}_{2}$, composed of abundant, inexpensive, and nontoxic elements. It finds use in several electromechanical devices, including resonators [1] in oscillator circuits and quartz crystal microbalances [2]. The highest frequencies that can be attained when using a quartz resonator as a frequency standard are limited by current industrial top-down methods of quartz production [3]. These often start with the hydrothermal synthesis of macroscopic quartz crystals, which has not evolved much in the recent decades [4,5,6]. After this, the crystals are machined, polished, and etched down to the desired resonator size. Until recently, this process has had a lower size bound of the order of a few micrometers, which in turn limited the natural resonator frequency below 1 GHz. In the last two decades, this has been pushed to the sub-micron range, achieving resonance frequencies in the GHz regime [7,8].

It is interesting to grow quartz as a thin film, bypassing all the downscaling and transfer steps and allowing lower resonator thicknesses. This has already been explored with chemical vapor techniques [9,10,11,12] and chemical solution methods [13,14,15,16,17]. Most recently, it was shown by Zhou et al. that GeO${}_{2}$ films can be grown with the the α-quartz structure homoepitaxially on single-crystal Al${}_{2}$O${}_{3}$ substrates by pulsed laser deposition [18].

Here we aim to grow quartz thin films on Si(100) substrates. The films are deposited first in the amorphous state and are crystallized following a post-annealing process. This route is chosen because it is not possible to crystallize amorphous silica films from the melt. The phase diagram of SiO${}_{2}$ imposes significant constraints: firstly, because silicon substrates melt at a lower temperature than SiO${}_{2}$ and, secondly, because other SiO${}_{2}$ phases are expected to become kinetically trapped [19,20]. A way around this constraint is to consider α-quartz isostructural materials with a lower melting point, such as GeO${}_{2}$, by itself or in solid solution with SiO${}_{2}$—an approach we followed in this work. The relative disadvantage of the lower abundance of GeO${}_{2}$ compared to SiO${}_{2}$ is somewhat compensated by its larger piezoelectric response [21]. In our films, the addition of GeO${}_{2}$ is motivated both by the larger piezoelectric response of this material, and by the easier handling of the GeO${}_{2}$-containing pellets compared to the more brittle SiO${}_{2}$ pellets.

In addition, a common strategy for lowering the process temperature is to weaken the strong silica network through the introduction of certain metal impurities. These are alternately referred to as devitrifying agents, melting agents, or network modifiers [22,23]. This has been successfully applied to various thin films [13,14,15,16,24,25], including amorphized layers on quartz [26,27,28] and GeO${}_{2}$ structures [29]. In this work, we introduce a Sr salt to the films to act as the melting agent.

## 2. Materials and Methods

For this study, we used Si(100) substrates, which were cut down to 15 × 15 mm${}^{2}$ squares from 4″ Czochralski-grown, boron p-doped (ρ∼1–10 Ωcm) wafers manufactured by Microchemicals GmbH (Ulm, (Baden-Württemberg), Germany). The cut substrates were washed with ethanol absolute, acetone, and isopropyl alcohol, as described in our previous work [30].

Thin films were grown using pulsed laser deposition (PLD) with home-made ceramic targets. The process was started by mixing SiO${}_{2}$ α-quartz (99.995%, 40 mesh), GeO${}_{2}$ (99.9999%), and SrCO${}_{3}$ (99.99%) powders, all purchased from Alfa Aesar (Haverhill, (MA), United States). We used an agate mortar and balls in a Fritsch Pulverisette ball mill (Fritsch, Idar-Oberstein, (Rhineland-Palatinate), Germany), rotating at 150 rpm for 30 min. The powders (2 g in total) were then recovered and pressed into a disk of 20 mm diameter and about 3 mm thickness under 10 tons in a hydraulic uniaxial press. The pellets were annealed in air at 900 ${}^{\circ }$C for 1 h. We used a UHV-capable vacuum chamber to house the PLD process. The samples were heated resistively and placed 5 cm away from the targets, which were ablated with a 248 nm KrF exciplex laser. For the ablation of these targets we used a spot size of 1.36 mm${}^{2}$ and a fluence of 2.5 J/cm${}^{2}$. The sample temperature during growth was kept to 100 ${}^{\circ }$C and the process pressure was 0.1 mbar O${}_{2}$.

After PLD growth, the samples were cut to 5×5 mm${}^{2}$ squares prior to annealing. The annealing step was carried out in an alumina crucible inside a Nabertherm tube furnace (Nabertherm GmbH, Lilienthal, (Lower Saxony), Germany), ramping at 20 ${}^{\circ }$C/min to 1000 ${}^{\circ }$C and maintaining this temperature for 5 h, powering down afterward (there were slight variations in the furnace temperature over time; for the 0%, 0.625%, 1.25%, 2%, and 3% samples (mole percent), a temperature setpoint of 1050 ${}^{\circ }$C was used as the equivalent of the original annealing conditions). A measurement of the cooling step, including the calculated cooling rate, is shown in Figure S1 of the Supplementary Material. The temperature of 1000 ${}^{\circ }$C was chosen on the basis of our previous research. Temperature-dependent GIXRD measurements in Figure S7 suggest that the onset of crystallization is close to 1000 ${}^{\circ }$C. The annealing was done under 200 cm${}^{3}$/min oxygen flow, at atmospheric pressure.

Coplanar grazing incidence X-ray diffraction (GIXRD) was used to characterize the crystalline films. For this work, we used a Panalytical X-pert Pro MRD thin film X-ray diffractometer (Malvern Panalytical, Malvern, (England), United Kingdom), with a 1/16${}^{\circ }$ divergence slit and a 4-bounce Ge(220) monochromator for the incident beam. We kept the incident angle of the primary beam on the sample to 0.55${}^{\circ }$, and we scanned only the 2θ angle. A PIXcel3D area detector (Malvern Panalytical, Malvern, (England), United Kingdom) was used in Scanning Line (1D) mode to improve counting statistics.

The topography of the samples, before and after film growth and crystallization, was analyzed with a Bruker Dimension Icon atomic force microscope (AFM) (Bruker, Billerica, (MA), United States). The microscope was used in tapping mode, with Tap300Al-G silicon probes from BudgetSensors (Sofia, Bulgaria), which have approximately a 40 N/m force constant and 300 kHz resonant frequency. Subsequent image correction included row alignment and background subtraction, using second-degree polynomials in both cases.

The optical microscopy images were captured with an Olympus Vanox-T microscope (Olympus Corporation, Tokyo, Japan). Images were auto white balanced during acquisition to correct for the lamp color temperature.

After crystallization, the surfaces were observed using scanning electron microscopy (SEM, Nova NanoSEM, FEI) (Thermo Fisher Scientific, Waltham, (MA), United States) combined with energy dispersive X-ray spectroscopy (EDS, Octane SDD detector by EDAX) (EDAX LLC, Mahwah, (NJ), United States) and electron backscatter diffraction (EBSD, using EDAX system equipped with Hikari Plus CCD camera) (EDAX LLC, Mahwah, NJ, United States). Team v.4.5, OIM Analysis v.8.1, and MTEX [31] v.5.4. softwarewere used to perform semi-quantitative elemental and crystal orientation analysis, respectively. MTEX is a free Matlab toolbox, and Team and OIM Analysis are both marketed by EDAX LLC, Mahwah, NJ, United States. Different acceleration voltages for the primary electron beams were used for EDS (5 kV) and EBSD (15 and 20 kV) in order to maximize depth and lateral resolution in the former, and provide a reasonable quality of Kikuchi patterns in the latter. EBSD observations were performed in low-vacuum mode (0.5 mbar of water vapor) to suppress charging and SEM image drift effects during the lengthy data collection. The texture analysis parameters were as follows: harmonic texture, using a harmonic series expansion (series rank 34), a Gaussian smoothing of 5.0 degrees, and triclinic sample symmetry.

XPS measurements were carried out in a UHV system (Omicron NanoTechnology, Taunusstein, (Hessen), Germany) with a background pressure below 10${}^{-10}$ mbar. The source was an Omicron XM-1000 monochromated Al Kα source. A pass energy to the detector of 50 eV was used. All scans were recorded with a step size of 0.1 eV and a time per step of 1 s. The elemental compositions were extracted by applying a Shirley background subtraction to the peaks and utilizing a Gaussian fit. XPS data analysis was carried out with CasaXPS v.2.3.19 (Teignmouth, (England), United Kingdom).

OriginPro 2018 b9.5.1.195 (Academic), by Originlab (Northampton, MA, United States), was used for general data visualization and line plots.

## 3. Results and Discussion

### 3.1. SiO${}_{2}$/GeO${}_{2}$ Film Growth

Five different targets, with a Si:Ge atomic ratio of 7:3 and varying amounts of SrCO${}_{3}$ (x), were used, with x = 0, 0.625, 1.25, 2, 2.5, 3, 5, 10, and 20 mole %. The 0% samples were grown as a control experiment. This concentration range is similar to that used by Zhang et al. in their recent CSD report [16].

The mixed SiO${}_{2}$/GeO${}_{2}$ films were all grown with the same parameters, described in Section 2. Because of the low growth temperature, the pristine films grew in the amorphous state. Supporting evidence of this can be seen in Figure S2.

The pristine films were characterized with AFM and XRR. Through the AFM results shown in Figure S3, we determined that these films were relatively rough (RMS roughnesses of 3–15 nm), containing visible particles with sizes in the few hundreds of nanometers, which probably originated from the target. We attribute this to the low thermal conductivity of SiO${}_{2}$ and GeO${}_{2}$ and/or poor target density, which all contribute to local heating and particle ejection [32].

XRR measurements of the films displayed only weak oscillations, possibly also as a consequence of the sample roughnesses. Nevertheless, the signal was enough to estimate the film thickness, which ranged between 120 nm and 210 nm for the SrCO${}_{3}$-containing films described here. The XRR analysis is detailed in the Supplementary Material, and the scans and fits are available in Figure S4, with an analysis of their critical angles in Figure S5.

### 3.2. Film Crystallization

**Figure 1:**
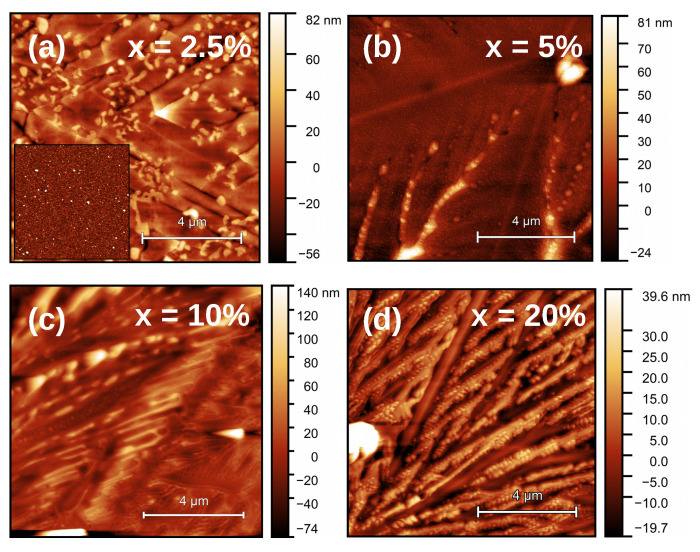
AFM pictures of the films after annealing. The RMS roughnesses are 18 nm (**a**), 8 nm (**b**), 12 nm (**c**), and 31 nm (**d**). Note that, while the image size is identical for all of them, the Z scale is not. Inset: image of the pristine 2.5% sample (RMS roughness = 3 nm).

**Figure 2:**
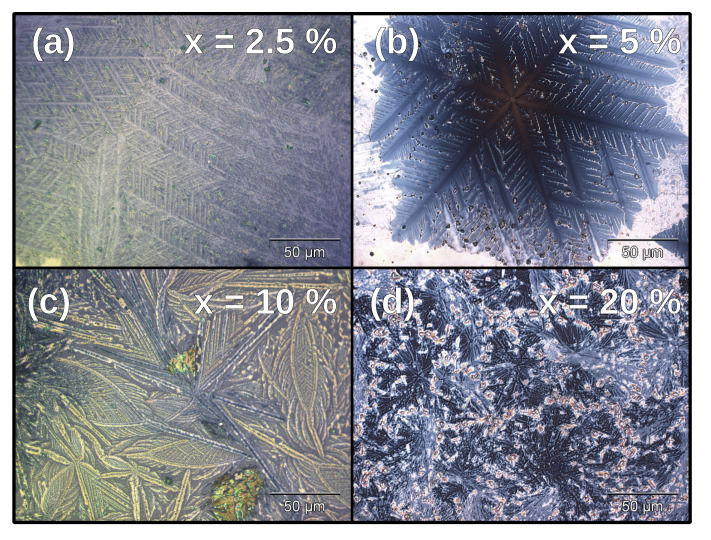
Optical microscopy pictures of SrCO${}_{3}$-containing Si${}_{0.7}$Ge${}_{0.3}$O${}_{2}$ samples after annealing. (**a**,**b**) The growth was dendritic at low Sr content. (**c**) The formation of long needles. (**d**) Small, disorderly crystalline features. Images of the remainder of the concentration series are available in Figures S2 and S8 of the Supplementary Material.

After annealing the PLD-grown films at 1000 ${}^{\circ }$C, they showed changes in topography. From the AFM measurements, we can observe the formation of micrometer-scale crystalline features in all SrCO${}_{3}$-containing films, as shown in Figure 1. The lateral size of some of these crystallites often exceeded the capabilities of our AFM (>40 μm). We note that the crystallization patterns changed depending on the films’ SrCO${}_{3}$ content. The differences in crystallization behavior became much clearer with optical microscopy, as seen in Figure 2. In films with x = 2.5% and 5%, dendritic crystallization appears to have taken place, while for x = 10% and 20% a less orderly pattern arises.

The elemental composition of the films was measured by X-ray photoelectron spectroscopy (XPS). We did this for x = 2.5 and 20% films, both before and after annealing. One could expect imperfect stoichiometry in the transfer of SiO${}_{2}$/GeO${}_{2}$ as a result of preferential ablation and differences in sticking coefficient [32], which would lead to Ge-poor films. Additionally, a loss of Sr might take place from the film bulk to the surface across the annealing process [13].

**Figure 3:**
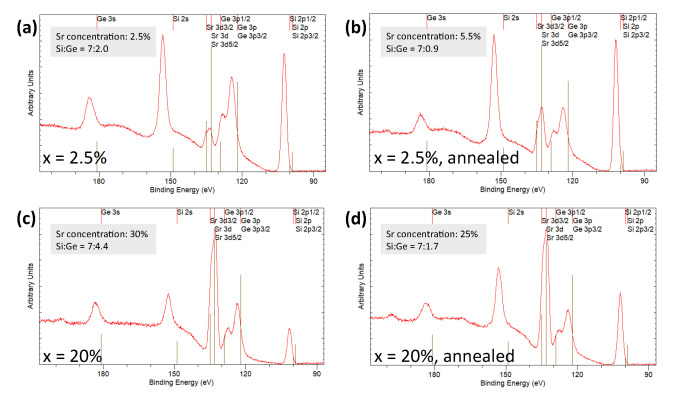
(**a**,**b**) XPS spectra of x = 2.5% samples before and after annealing. (**c**,**d**) XPS spectra of x = 20% samples before and after annealing. The grey insets show the concentrations of Sr and the ratios of Si to Ge that were extracted from the spectra. We estimate the error in the individual elemental amounts to be ±0.5%.

As the results in Figure 3 indicate, the Si:Ge ratio was indeed higher than the one used in target synthesis (i.e., 7:3) in all but the first panel, which corresponds to the as-grown x = 20% sample. The rest were consistent with the preferential Si transfer. After annealing, the ratio was further increased, which we attribute to the higher volatility of Ge compared to Si in the oxide matrix. The as-grown Sr atomic content was consistently larger than expected for the x = 20% sample, and it decreased after annealing. In contrast, the Sr atomic % was correct within error for the as-grown x = 2.5% sample, but it nearly doubled after annealing.

**Figure 4:**
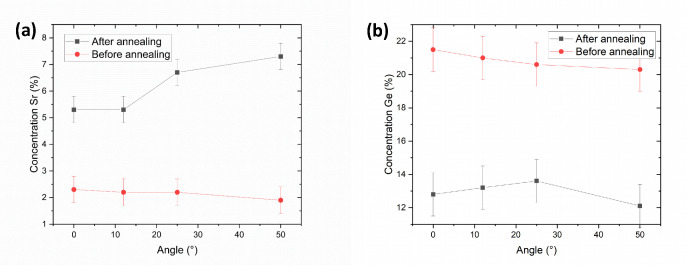
Angle-dependent XPS-determined elemental concentrations of the x = 2.5% samples before and after annealing. The Sr and Ge concentrations are shown in (**a**,**b**) respectively. The angle is defined with respect to the surface normal of the samples.

We know from the work by Carretero et al. that annealing Sr-doped SiO${}_{2}$ films causes the Sr to be expelled to the film surface as crystallization takes place [13]. Hence, a decrease in the film Sr content was expected. However, the increase of Sr content for the x = 2.5% sample seems, at first, to contradict such behavior. We therefore performed angle-dependent XPS in order to gain more information about the elemental composition of the x = 2.5% films as a function of depth. We show in Figure 4a that, after annealing, the Sr content increased across all measured angles, becoming highest at larger angles, which indicates an accumulation closer to the sample surface, in agreement with Carretero et al. [13]. Figure 4b further shows that while annealing lowered the Ge content as well, it no longer had an angle dependence within error. We are limited here by the probing depth of XPS (which is about 5 nm at normal incidence and decays with the cosine of the incident angle), though it stands to reason that the film was Sr-depleted further from the surface. In the case of the annealed x = 20% film, annealing seemed to reduce the Sr concentration at normal incidence, rather than increasing it.

Specular 2θ/ω XRD scans are largely devoid of any signal originating from the film (see Figure S6 of the Supplementary Material), with only the substrate multiple diffraction peak near 33${}^{\circ }$ appearing reliably. Importantly, while 2θ/ω scans of the samples do not show Bragg peaks from oriented phases, oriented crystalline areas with sizes smaller than the coherent length of the X-rays could still be present.

**Figure 5:**
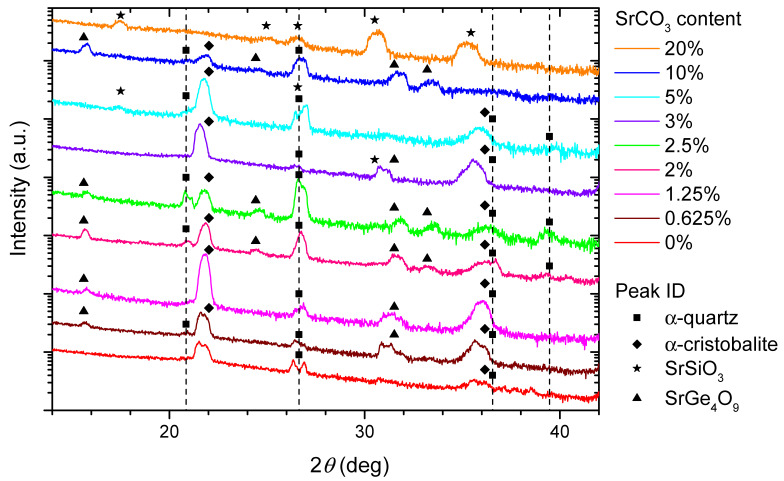
GIXRD scans of the annealed sample series, in the 2θ range of 14–42${}^{\circ }$. The vertical lines denote the position of diffraction peaks from the α-quartz structure [33]. The full scan (10–80${}^{\circ }$) is shown in Figure S9.

GIXRD measurements are shown in Figure 5. A version of this figure with an extended 2θ range is available in Figure S9 of the Supplementary Material. In that version, it is most noticeable that all scans show a characteristic broad feature near 55${}^{\circ }$. This peak is also present in films with no SrCO${}_{3}$, and it can also be seen when measuring non-annealed films and pristine Si(100) substrates. The source of this signal, which we reason to be from the Si substrate, is further discussed in the Supplementary Material (Figure S10).

Besides this signal, we identify several diffraction peaks from the films which are consistent with the presence of α-quartz [33] and α-cristobalite [34]. The α-cristobalite peaks are more widely present than those of α-quartz, appearing in all samples with Sr content between 0 and 10%. The relative fraction of the α-quartz phase was largest for the 2.5% sample. Using the integrated peak area for α-quartz and α-cristobalite low-angle reflections, we roughly estimate the quartz-to-cristobalite molar ratio to be 1.9 in the x = 2.5% sample, and 0.6 in the x = 2% sample, which has a visibly weaker quartz peak.

There are also other peaks, most obvious below 20${}^{\circ }$, which do not belong to the common SiO${}_{2}$ phases, but most likely to strontium silicate and strontium tetragermanate. Specifically, the peak at 17.4${}^{\circ }$ for x = 20% is close to the position where we would expect to see signal in the case of the (002) peak of monoclinic SrSiO${}_{3}$ (strontium metasilicate [35]). Using this as a starting point, we were able to assign all the peaks in the x = 20% scan to the strongest reflections of this one Sr compound. We saw the same phase in crystallization experiments of SiO${}_{2}$/GeO${}_{2}$ multilayers grown by ALD (see Figure S11 of the Supplementary Material and Reference [30] for growth details). Some of these peaks are also present for x = 5%, albeit much weaker.

With the exception of the 3% film, the remainder of the strontium-containing samples show a weak diffraction peak near 15.6${}^{\circ }$. This peak is accompanied by others, including those at 31.6${}^{\circ }$ and 33.5${}^{\circ }$, which we cannot assign to strontium silicate. However, similar features have been observed recently on GeO${}_{2}$ films grown on SrTiO${}_{3}$ substrates [18], and they were assigned to a strontium germanate phase. We therefore conjecture that the diffraction peaks in our spectra belong to crystalline SrGe${}_{4}$O${}_{9}$ (strontium tetragermanate [36], trigonal), which matches quite well most of the observed peaks, even though we would expect a stronger signal near 24.4${}^{\circ }$.

The x = 0% scan shows weak peaks for α-quartz and α-cristobalite. Our GIXRD measurements do not, in principle, allow us to quantitatively compare the amount of crystalline phases in different samples, but AFM images of the 0% sample (Figure S2 in the Supplementary Material) reveal no obvious signs of crystallization, which hints at a much smaller crystal size than in the SrCO${}_{3}$-containing samples. This discrepancy between micrographs and GIXRD spectra can also arise if the crystal growth begins close to the substrate–film interface, in which case it would not be immediately visible in AFM.

From GIXRD results alone, a trend was not found across the sample series. The 20% sample displays SrSiO${}_{3}$ peaks only. The remaining Sr-containing samples show a less-straightforward behavior, with the SrGe${}_{4}$O${}_{9}$ and SrSiO${}_{3}$ peaks vanishing and reappearing along the series. We know that phases with uniformly random orientation are overrepresented in GIXRD with respect to oriented phases, and thus Figure 5 shows an incomplete picture. Therefore, in order to obtain more reliable information about the crystallinity of different samples in the series, we performed EBSD measurements.

The 20% sample shows electron diffraction patterns at certain points, but they do not belong to α-quartz. With this, we have no evidence of a crystalline silica phase in this sample, which suggests that the high Sr content in this sample resulted in the preferential formation of Sr compounds such as SrSiO${}_{3}$. We note that the weakly diffracting domains appeared to be spherulitic [18,37]. Figure S12 of the Supplementary Material shows the topography and composition maps of this sample.

Lowering the Sr content, the most noticeable features of the 10% sample were long needles (see Figures S13 and Figure S14 in the Supplementary Material), which also yielded diffraction patterns corresponding neither to α-quartz nor α-cristobalite. However, patches of α-quartz could be found elsewhere on the sample surface. Therefore, we conjecture that the Sr concentration can locally be low enough to preclude the formation of silicate or germanate crystals, but still high enough to promote α-quartz crystallization in selected locations.

**Figure 6:**
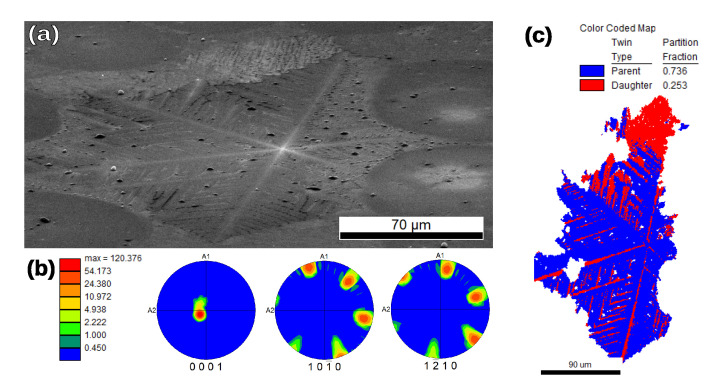
EBSD analysis of the x = 5% sample. Indexing is made with the α-quartz unit cell. (**a**) 71${}^{\circ }$-tilted SEM image of one of the dendrites. (**b**) Orientation distribution function (ODF) plotted for three different poles in multiple of random distribution (MRD) scale indicates a single crystal with [c] direction almost perpendicular to the sample surface (A3 axis). (**c**) EBSD map of the dendrite from (**a**), whose lateral size is a few hundred micrometers. Map demonstrates the presence of two Dauphiné twin-related orientations inside the dendrite. No other grain boundaries (>3${}^{\circ }$ misorientation between neighboring pixels) are present.

The 5% sample showed dendritic crystals, as seen in Figure 2b and Figure 6. EBSD analysis reveals that these crystals were made of α-quartz, and that the area around them did not produce any diffraction pattern. Some Dauphiné twinning [38] was observed in the dendrites, but the orientation was otherwise close to uniform within the twins, with only very small lattice rotation being present. Dauphiné twinning in α-quartz is detrimental to piezoelectric properties, and therefore to performance in many applications. Faster cooling through the β-to-α-quartz transition may reduce the extent of this twinning [38].

The dendrites had six-fold symmetry (in agreement with the α-quartz structure) and their arms’ longitudinal axes coincided with the <a> directions, 〈11$\bar{2}$0〉. In some cases, we observed two dendrites sharing a center and growing outward with different orientation, resulting in the apparent growth of dendrites with more than six arms.

**Figure 7:**
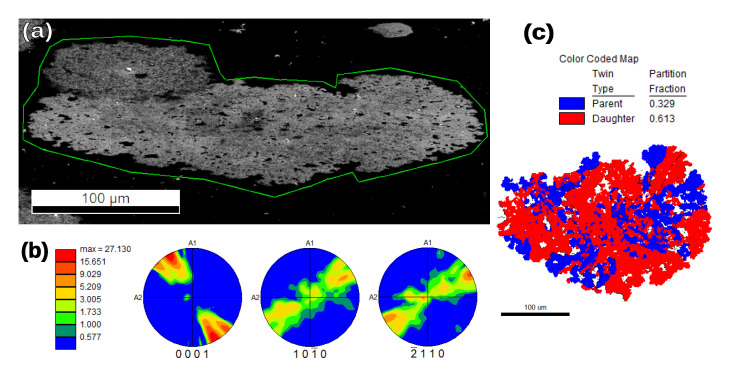
EBSD analysis of a x = 2.5% sample. Indexing is made with the α-quartz unit cell. (**a**) 71${}^{\circ }$-tilted SEM image of a crystalline, non-dendritic region. (**b**) ODF plotted in three different pole figures. The [0001] figure shows a certain orientation, tending to a single in-plane direction. (**c**) EBSD map of the region, showing that the entire formation is 200–300 μm in size; the twinned regions are smaller. There are some grain boundaries present (black lines). The distribution of Dauphiné twins is more disorderly than in the dendritic areas.

In the 2.5% sample, we observed the formation of quartz dendrites very similar to those in the 5% sample. Interestingly, there were also non-dendritic crystalline α-quartz regions. These contained some grain boundaries and many Dauphiné twin boundaries. One of these regions is shown in Figure 7.

The main distinction between the dendritic and non-dendritic formations can be made on the basis of their respective ODFs (see Figure 6b and Figure 7b). These show that a region identified as a single dendrite (Figure 6) had close to uniform orientation. In particular, all the dendrites that we analyzed had their *c* crystal axis closer to the film normal than to the film plane. For the non-dendritic growth, it is apparent that, within a single grain formation, there can be preferred orientation for the *c* axis with relatively large local misorientations inside one grain (Figure 7b). When extending the measurement to include several spherulites, the *c* axis shows a rather random orientation (see Figure S15 of the Supplementary Material).

It is possible that the differences in crystallization mode were caused by inhomogeneities in the Sr concentration in the film. We propose that areas with larger concentrations of melting agent had their devitrification onset at lower temperatures. This would result locally in a larger supercooling in regions with lower Sr concentration. We know that increased supercooling leads to spherulites forming preferentially over single crystals [37,39]. Therefore, a nonuniform distribution of Sr impurities can result in local variations in the crystal growth mode.

In this complex material, symmetric 2θ/ω scans, GIXRD, and EBSD together are needed to give insights about the crystallinity of the films. The lack of features in specular 2θ/ω is an indicator that no strong out-of-plane texture was present in any of the films. GIXRD scans show some α-quartz and α-cristobalite signal in the control (0% SrCO${}_{3}$) sample after annealing, which suggests that temperature alone was enough to inducesome crystallization.

The GIXRD cristobalite signal clearly increased with the addition of SrCO${}_{3}$, only decreasing at 10% SrCO${}_{3}$. The presence of quartz peaks in GIXRD was also determined by the devitrifying agent, with these peaks becoming more intense for intermediate concentrations. The 20% sample does not show any silica peaks, either from quartz or cristobalite. We show that SrCO${}_{3}$ did act as a devitrifying agent and promoted the crystalline silica phases, but in large amounts formed silicates and germanates instead. This is supported by the EBSD observations of the x = 2.5% and 5% samples, which showed 100-μm scale α-quartz structures with varying degrees of orientation and twinning. Only α-quartz, and no α-cristobalite, was found by EBSD. This discrepancy with the X-ray diffraction results is most likely due to the surface sensitivity of EBSD. Optical microscopy (Figure 2 and Figure S8) confirmed the formation of microscopic crystals for Sr concentrations above 0.625%. The 3% sample is anomalous in both GIXRD and optical microscopy measurements (see Figure 5 and Figure S8)). Its behavior, more similar to that of samples with x < 2%, could be explained by a strongly inhomogeneous Sr distribution across the film.

We therefore note that low concentrations of SrCO${}_{3}$ were required for the crystallization of partially oriented α-quartz, and in order to avoid the formation of strontium silicates or germanates. It is also clear from our observations that the crystallization behavior was not uniform across the sample surface (∼5 × 5 mm${}^{2}$), with the 2.5% sample showing both dendritic *and* spherulitic growth. This was most likely due to inhomogeneous distribution of Sr during film growth. This could be solved in the future by growing similar structures using atomic layer deposition (ALD) as multilayers of SiO${}_{2}$ and GeO${}_{2}$ [30], including intermediate layers of SrO.

Barring the highest SrCO${}_{3}$ concentrations, we were able to crystallize silica phases. As the interest is in piezoelectric properties, it is desirable to grow α-quartz preferentially over α-cristobalite. The growth of α-cristobalite, while indicative in part of the effectiveness of the devitrifying agent, competes directly with the growth of α-quartz. In order to avoid the cristobalite phase, lower temperatures might be required. This may be challenging, as similar experiments of pure GeO${}_{2}$ films on sapphire substrates suggest that lowering the temperature can result in spherulitic (rather than dendritic) α-quartz formation [18]. Nevertheless, the appearance of the two competing silica phases in the XRD spectra, together with the nonuniform crystallization behavior observed in optical and electron microscopy (including EBSD), clearly sets the requirement for a better method to distribute the SrCO${}_{3}$ impurities throughout the film.

The crystallization behavior that we observed in our samples makes them especially difficult to characterize with a single technique. EBSD is an excellent tool to analyze the sample orientation at a local scale, but it is surface-sensitive and has difficulty detecting crystallites which are covered in amorphous oxide. Our XRD scans were able to detect phases comparatively deeper in the sample, but they require uniformly random orientation (GIXRD) or strong texture (specular 2θ/ω). Particularly in the case of the large quartz formations that we found (Figure 6 and Figure 7), they fell into an intermediate regime, and thus were only clearly visible with EBSD. The cristobalite phase that appeared in most of the samples described in this manuscript was either present in very small crystallites or buried under other phases, and therefore only present in GIXRD scans. Future work with this type of sample will require an experimental technique that can efficiently overcome some of the limitations mentioned so far. One such technique could be micro XRD [40,41], which has the ability to penetrate deeper into the film, and—provided that the crystallite size is large enough—could permit local (if complicated) phase and orientation analysis, allowing efficient mapping of the samples’ crystallization behavior.

## 4. Conclusions

We demonstrated the efficacy of SrCO${}_{3}$ as a melting agent to trigger SiO${}_{2}$/GeO${}_{2}$ crystallization on silicon at accessible process temperatures. We showed that dendritic α-quartz could grow at small SrCO${}_{3}$ concentrations of around 2–2.5%, avoiding the appearance of SrSiO${}_{3}$ and SrGe${}_{4}$O${}_{9}$, which became the prevalent phases when the melting agent reached 10% and 20% atomic concentrations. We observed a certain heterogeneity in crystal formation at different points of samples, which suggests that a more uniform distribution of the melting agent is necessary.

## Data Availability

The data presented in this study are available on request from the corresponding author.

## Supplementary Materials

The following are available at https://www.mdpi.com/article/10.3390/nano11071654/s1, Figure S1: Plot of temperature and cooling rate of the tube furnace used during the annealing and cooling process, as a function of time. The temperature closely follows a double exponential decay, and as a consequence, so does the cooling rate, Figure S2: (a) AFM images of a sample without Sr before annealing (RRMS = 21 nm). (b) AFM after annealing (RRMS = 21 nm). (c) GIXRD scans of both the as-deposited and annealed 0% samples, together with a pristine 2.5% sample for comparison, Figure S3: AFM pictures of the pristine films. The RMS roughnesses are 3 nm (a), 15 nm (b) 4 nm (c), 7 nm (d), Figure S4: XRR scans (solid lines) and fits (dotted lines) for samples with x = 0–20% grown with 1800 laser pulses. The legend shows the SrCO_3_ content as well as the thickness rendered by the fit, Figure S5: Plot of the critical angle for total external reflection of the sample series. The values calculated from the bulk densities (assuming stoichiometric transfer and additive volumes of Si_0.7_Ge_0.3_O_2_ and SrCO_3_) are shown in black and the experimentally-determined values are in red. The error bars are based on the width of the Gaussians used to obtain the experimental values, which is larger than the scan step size for all measurements, Figure S6: Symmetric 2q/w scans for the film series. The multiple diffraction signature is visible near 33${}^{\circ }$. In the 2% scan, weak reflections from SrGe_4_O_9_ are visible above the noise, Figure S7: GIXRD scans at various temperatures for a sample containing 10% SrCO_3_. The peak near 24${}^{\circ }$ corresponds to the graphite dome over the sample, and the weaker peak near 21.7${}^{\circ }$ most likely belongs to an a-cristobalite phase. Note that this peak is at a lower angle at high temperatures due to the thermal expansion of the material, Figure S8: Optical microscopy pictures of SrCO_3_-containing samples after annealing. (a) The surface is decorated with particles in the few micron size range, but no appreciable crystals. (b) Micron-sized crystals are apparent locally, whereas most of the surface is covered in circular features. (c) Crystals of irregular geometry cover large areas of the sample. (d) Some branched crystalline areas appear sporadically on the surface, Figure S9: Extended GIXRD scans, Figure S10: (a) Simulated pole figure for the (113) reflections of a Si(100) substrate. Note that this is a polar plot, with c in the radial direction (equidistant ticks) and f in the circumference, increasing counter-clockwise. Red squares indicate poles in the upper hemisphere, and blue boxes are for poles in the lower hemisphere, thus outside the measurement range. For Si(100), the projections overlap for the upper and lower hemispheres. Simulation details available at Reference 7. (b) GIXRD scans taken on a sample on Si(100) at various f rotation values. The sample was realigned prior to each scan. The red rectangles highlight the signal near 56${}^{\circ }$, which is present in the 0${}^{\circ }$, 90${}^{\circ }$, 180${}^{\circ }$, and 270${}^{\circ }$ scans, Figure S11: Scanning electron microscopy characterization of crystal domains formed after crystallization of a SiO_2_/GeO_2_ ALD multilayer into SrSiO_3_ (monoclinic9). (a) Secondary electron image; (b) Forward scattered electron image of area studied by EBSD; c) [001] IPF+IQ map showing the crystal direction parallel to the sample surface normal; (d) (001), (010) and (100) sample texture plots using scale of multiples of random distribution (MRD); (e) example of Kikuchi pattern and (f) corresponding crystal orientation (axonometric projection in top view), Figure S12: (a) SEM image of a x = 20% annealed film. Annealing has a clear effect on sample topography, which suggests the growth of spherulites whose crystallinity we were not able to ascertain. (b–e) EDS composition maps for the main elements in the films. From these maps, it appears that the grain boundaries are Si-depleted, which can be attributed to increased sample thickness in those regions. EDS is sensitive to signal from the Si substrate in the case of relatively thin films, and likewise if a part of the film is thicker, EDS will display proportionally higher contributions from elements in the film (which includes Si, but at a much lower atomic percent than the substrate does), Figure S13: EBSD analysis of the x = 10% sample of the series. Indexing is made with the a-quartz unit cell. (a) 71${}^{\circ }$-tilted SEM image of a region containing both crystalline needles and spherulites. The red rectangle shows the area analyzed with EBSD. (b) ODF plotted in three different pole figures. The intensity scale is in multiples of random density (MRD). (c) [001]-IPF (Inverse Pole Figure) of the region. There is no preferred orientation overall, but the patterns show that there are quartz regions in addition to the non-quartz, long needles, which we have been unable to index, Figure S14: (a) SEM image of an x = 10% annealed film. After annealing, needles (in light grey) of length in the tens of micrometers become visible. In regions of this type, EBSD indicates that there are no crystalline SiO_2_ phases, therefore the needles might correspond to a silicate or germanate phase. (b–e) EDS composition maps for the main elements in the films. The needles appear to be not only silicon poor (which can be explained by their added thickness, see Figure S12), but also oxygen defficient, Figure S15: EBSD analysis of the x = 2.5% sample of the series, including multiple coagulated islands. Indexing is made with the a-quartz unit cell. We have not been able to index the region between the islands. (a) 71${}^{\circ }$-tilted SEM image of a crystalline, non-dendritic region. (b) ODF plotted in three different pole figures shows the presence of a few grains (∼5) with different orientations. (c) EBSD map of the region showing parent and daughter twinned areas. Grain boundaries indicated with black lines separate islands with different crystal orientations.
