# Supplementary material for: Growth and Crystallization of SiO2/GeO2 Thin Films on Si(100) Substrates
Source: Nanomaterials (Basel). 2021 Jun 23;11(7):1654. doi: 10.3390/nano11071654 (PMC8305750; doi:10.3390/nano11071654)
Supplement: Supplementary file 1 [file nanomaterials-11-01654-s001.zip › nanomaterials-1265768-supplementary.pdf]

# Supplementary Material: Growth and crystallization of $SiO_2/GeO_2$ thin films on Si(100) substrates

Jordi Antoja-Lleonart,[1] Václav Ocelík,[1] Silang Zhou,[1] Kit de Hond,[2] Gertjan Koster,[2] Guus Rijnders,[2] and Beatriz Noheda[3, a)]

[1)]*Nanostructures of Functional Oxides, Zernike Institute for Advanced MaterialsUniversity of Groningen, 9747AG Groningen, The Netherlands*

[2)]*MESA+ Institute for Nanotechnology, University of Twente, PO Box 217, 7522NH Enschede, The Netherlands*

[3)]*Nanostructures of Functional Oxides, University of Groningen, 9747AG Groningen, The Netherlands*

(Dated: 23 June 2021)

## I. FURNACE COOLING RATES

Figure S1 shows a measurement of the cooling process (from 1050ºC) which followed the film annealing. The data was taken on the same tube furnace that we have used for the annealing processes in this work. The cooling is passive, and it starts by powering off the heating elements around the tube, hence the cooling rate increases markedly in the first few datapoints and tapers off afterward. The temperature- and time-resolution of the temperature plot are, respectively, ±1ºC and ±1 min.

## II. CONTROL SAMPLES

In Figure S2 we include AFM images and GIXRD patterns of thin films that did not contain any $SrCO_3$ (see Reference 1 for a similar control). Although large particles remain visible in spots, the RMS roughness appears not to change through annealing. In some regions, AFM clearly shows the appearance of holes in the sample, which we attribute to bubbles formed due to Ge or O loss (see Reference 2). In the GIXRD scans of annealed Sr-less samples, there are small peaks below 30º which we have attributed to $\alpha$-cristobalite and $\alpha$-quartz. Figure S2 also shows the GIXRD spectrum for an as-grown x = 2.5% sample, confirming the absence of film diffraction peaks before the anneal step *including* in samples that do contain $SrCO_3$.

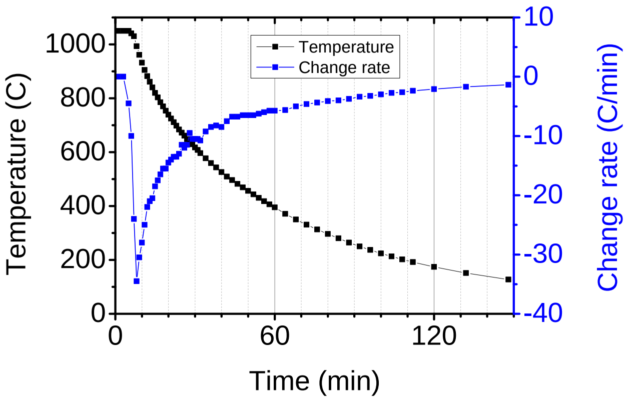


FIG. S1. Plot of temperature and cooling rate of the tube furnace used during the annealing and cooling process, as a function of time. The temperature closely follows a double exponential decay, and as a consequence, so does the cooling rate.

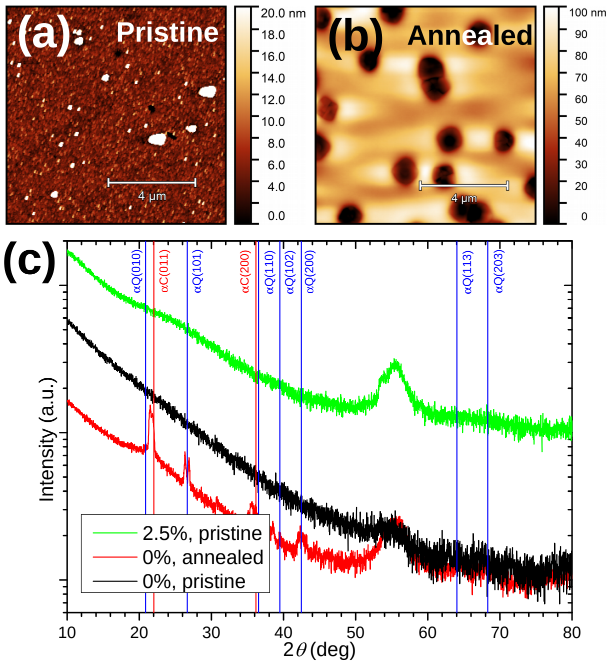


FIG. S2. a) AFM images of a sample without Sr before annealing ($R_{RMS}$ = 21 nm). b) AFM after annealing ($R_{RMS}$ = 21 nm). c) GIXRD scans of both the as-deposited and annealed 0% samples, together with a pristine 2.5% sample for comparison.

## III. AFM MEASUREMENTS OF PRISTINE SAMPLES

Figure S3 shows AFM topography (height) data for four pristine samples of varying Sr concentration. Particles of similar size are visible in all of them, but no crystallization is apparent, unlike the images of their annealed state in Figure

[a)]Electronic mail: b.noheda@rug.nl

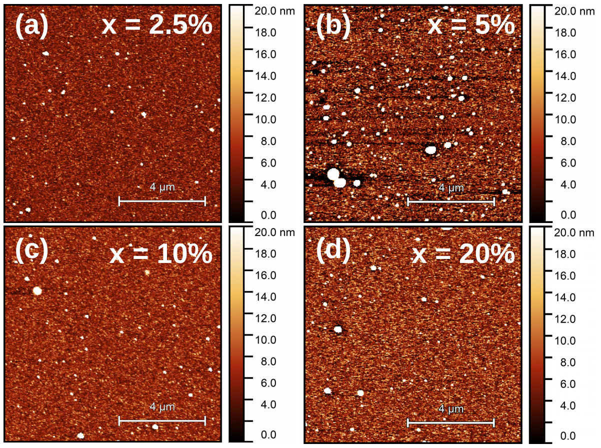


FIG. S3. AFM pictures of the pristine films. The RMS roughnesses are 3 nm (a), 15 nm (b) 4 nm (c), 7 nm (d).

1.

## IV. X-RAY REFLECTOMETRY

We performed X-ray reflectometry (XRR) measurements of the pristine PLD-grown samples for an indirect estimation of the film thicknesses. The results are shown in Figure S4.

Our measurement system is a Panalytical X-pert Pro MRD thin film X-ray diffractometer, with a 1/16° divergence slit and a Ge(220) hybrid monochromator for the incident beam. The detector is a proportional counter fitted with a 1/8° slit.

Likely because of the interface roughness of the films, the fringes in some of our scans are rather weak. We have overcome this problem by measuring thinner films, and then extrapolating the growth to the films we used for annealing. The XRR scans shown here correspond to films grown with 1800 laser pulses. The films used for annealing in the main text were grown with twice as many laser pulses, 3600, and thus we expect that they will be approximately twice as thick.

We used the GenX software to fit the experimental data[3]. In Figure S4 we show the XRR scans together with their fits, and the $SiO_2$/$GeO_2$ layer thicknesses are shown for each sample. For the aforementioned reasons, the results are approximate at best, and we use them only as an estimate for the film thicknesses. We have also extracted the critical angle values for each of those scans by differentiating the intensity and fitting the area near the critical angle with a Gaussian function. For each case, the calculated value (from the theoretical density and composition of the target) is compared to the center of that Gaussian in Figure S5. The error in the critical angle data is comparable to the differences between the samples, which makes it difficult to draw strong conclusions. However, it can safely be said that the experimental critical angle values are generally lower than expected from our calculations.

The calculated mass density and the scattering length density (SLD) of the films both increase with $SrCO_3$ following approximately linear trends. Translating the critical angle data to SLD (SLD $\propto \theta_c^2$), we can say that, past x = 5%, the experimental SLD also increases with $SrCO_3$ concentration, although the increase seems to be more dramatic in the measured data. In glassy $GeO_2$ containing alkali ions, it has been observed that density has an anomalous dependence on increasing alkali concentration, showing a maximum around x = 15-20%[4]. This increase occurs due to a progressive change of the Ge atom coordination. Although this anomaly may not apply equally to Sr ions, a similar behavior could explain the disparity between the experimental results and our predicted data, which is a weighted average of the bulk densities for $SiO_2$, $GeO_2$ and $SrCO_3$, therefore not capturing the details of possible structural changes in the silica-germania matrix.

## V. SPECULAR 2$\theta$ SCANS

Figure S6 shows the specular $2\theta/\omega$ measurements done on six annealed samples of different Sr content. These were recorded in the same setup as the GIXRD scans, only adding a 1/16° slit and changing the area detector to 0D (receiving slit) mode. The only clear feature of these scans is a Si diffraction peak near 33°, which is often visible in specular measurements on Si(100) substrates, regardless of the presence and nature of thin films grown on them. This peak occasionally gets mislabeled as Si(002), which itself is a forbidden reflection with F *exactly* equal to zero[5]. Instead, the peak we observe originates from multiple scattering in the Si lattice, and its intensity varies strongly with misalignment in $\phi$ (which is further evidence that it does not belong to a plane parallel to the sample surface), thus it is not identical in all five scans. With the exception of the 2% sample (where some peaks appear that we have already attributed to $SrGe_4O_9$), no film signal appears in the scans, which suggests that either the crystalline phases are very minoritary or they are not suitably oriented for specular measurements, both of which could reduce their associated peak intensity below the shot noise.

## VI. GIXRD HEATING RUN

We were able to measure GIXRD scans of the samples while heating them in an Anton Paar DHS1100 stage under an oxygen-rich atmosphere. The scans in Figure S7 only show the randomly oriented fraction of the crystalline oxide, which is why we cannot easily rule out the onset of crystallization being at a lower temperature. The results of one such heating experiment are shown in Figure S7. Based on the scans, we see the first signs of a crystalline $SiO_2$ phase appearing at 1000ºC.

## VII. FULL GIXRD SCANS AND GIXRD FEATURE

In Figure S9, we show the complete GIXRD scans from which Figure 5 of the main text has been extracted. The GIXRD scans for many of the samples reported here show

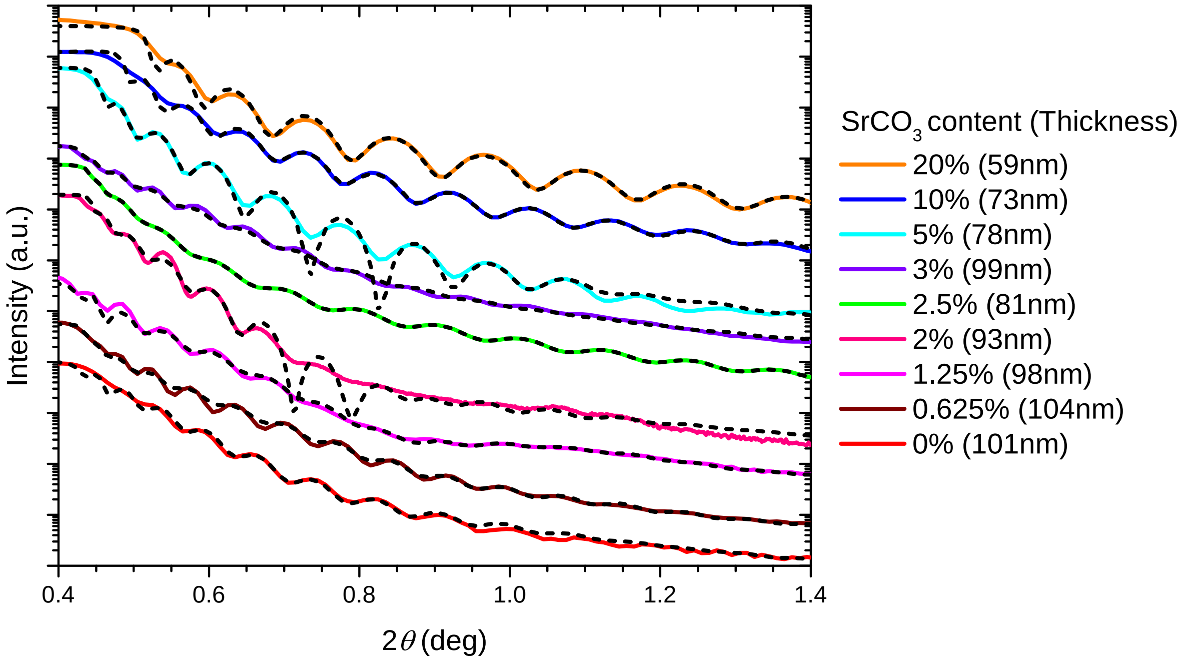


FIG. S4. XRR scans (solid lines) and fits (dotted lines) for samples with x = 0-20% grown with 1800 laser pulses. The legend shows the $SrCO_3$ content as well as the thickness rendered by the fit.

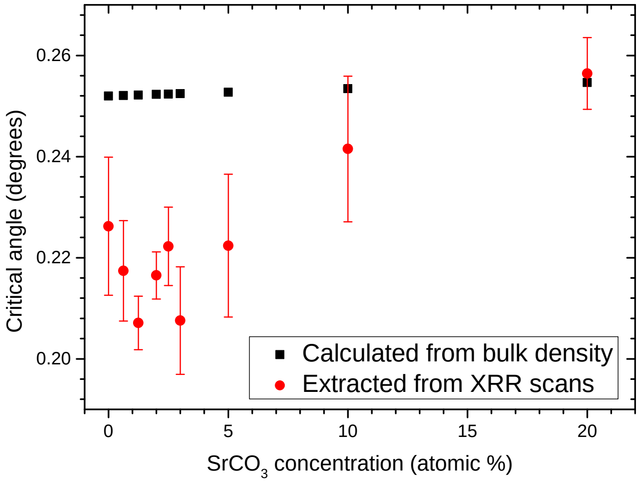


FIG. S5. Plot of the critical angle for total external reflection of the sample series. The values calculated from the bulk densities (assuming stoichiometric transfer and additive volumes of $Si_{0.7}Ge_{0.3}O_2$ and $SrCO_3$) are shown in black and the experimentally-determined values are in red. The error bars are based on the width of the Gaussians used to obtain the experimental values, which is larger than the scan step size for all measurements.

a characteristic bump near 56°. Although scantily reported, this feature has been observed by other authors, who conjecture that the peak is from the Si(113) family[6]. GIXRD of a pristine Si(100) substrate also reveals this same signal, which means that it does not originate in the PLD-grown film and that it is not a result of annealing. As shown in Figure S10,

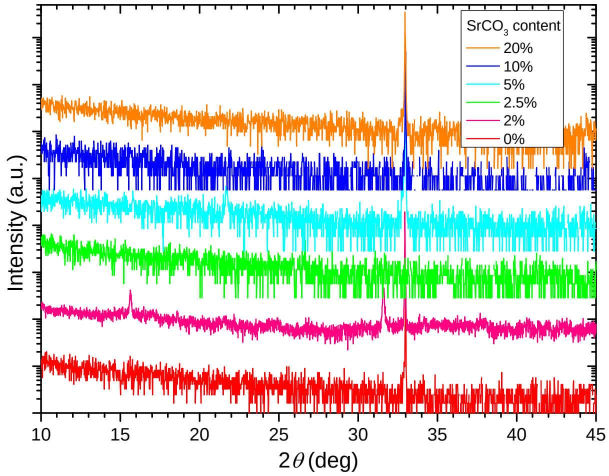


FIG. S6. Symmetric $2\theta/\omega$ scans for the film series. The multiple diffraction signature is visible near 33°. In the 2% scan, weak reflections from $SrGe_4O_9$ are visible above the noise.

we have simulated a pole figure of the Si(113) plane family (whose Bragg peaks we expect at $2\theta = 56.13°$) for the case of a Si(100) substrate, with its [011] axis at 0°. There are, among others, four poles at $\chi$ near 25.24° and $\phi$ = (0+90n)°. During the XRD measurements shown here, the substrate edges (along their [011] direction) are approximately parallel to the beam, which means that two of these poles are at, or very close to the scattering plane during the XRD scans shown in Figures 5 and S2. During a GIXRD scan, these planes come near the diffraction condition. Weiss reports an additional, much

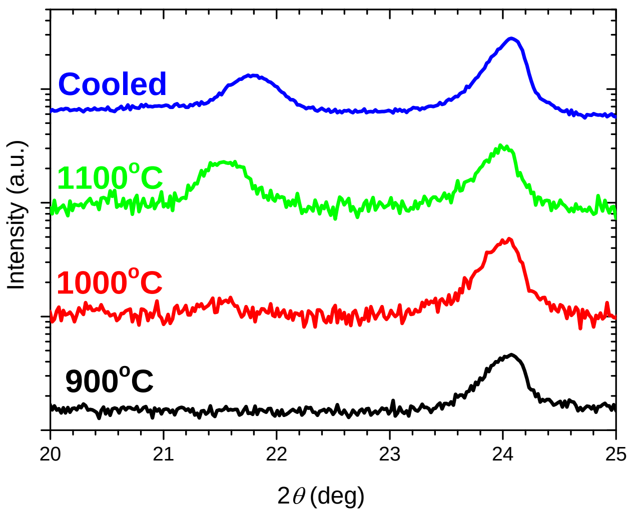


FIG. S7. GIXRD scans at various temperatures for a sample containing 10% $SrCO_3$. The peak near 24° corresponds to the graphite dome over the sample, and the weaker peak near 21.7° most likely belongs to an $\alpha$-cristobalite phase. Note that this peak is at a lower angle at high temperatures due to the thermal expansion of the material.

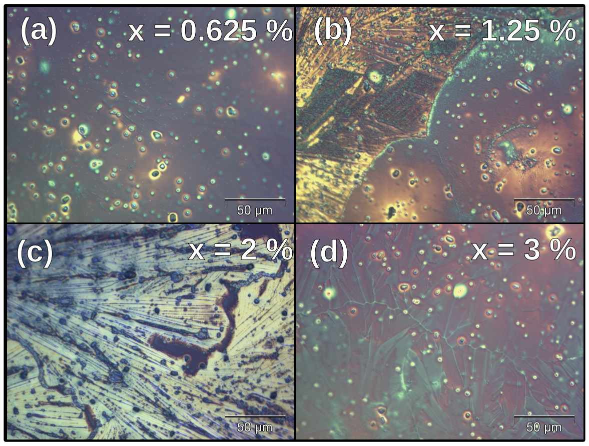


FIG. S8. Optical microscopy pictures of $SrCO_3$-containing samples after annealing. a) The surface is decorated with particles in the few micron size range, but no appreciable crystals. b) Micron-sized crystals are apparent locally, whereas most of the surface is covered in circular features. c) Crystals of irregular geometry cover large areas of the sample. d) Some branched crystalline areas appear sporadically on the surface.

sharper peak near 51°, which is precisely where it should be expected if we account for the incident angle being nonzero[6] and including the $\chi$ angle reported in Figure S10. However, 51° lies several degrees off of the expected $2\theta$ value from the Si(113) plane spacing. Conversely, the signal at 56° has the correct position in $2\theta$ but the wrong orientation in $\chi$, and it is tentatively explained by Weiss as a consequence of beam divergence.

We have aditionally performed GIXRD while rotating a sample along the $\phi$ direction, shown in Figure S10, which reveals that this signal appears, sometimes weakly, at multiples of $\phi$ = 90° and vanishes otherwise. This shows with more conclusiveness that, even though it appears in a GIXRD scan, this signal does not come from a randomly oriented phase, and agrees with the Si(113) origin hypothesis. Due to the imperfect match between $2\theta$ and $\chi$ for this reflection, its intensity is diminished, bringing it to approximate parity with thin film reflections.

## VIII. $SIO_2/GEO_2$ CRYSTALLIZATION TEST

The distribution of Sr species in the film seems to play an important role during crystallization. In our earlier work[8] we described the ALD growth of $SiO_2/GeO_2$ multilayers with varying degrees of intermixing.

We used a PLD system to grow metallic strontium particles (from a target made out of dendritic Sr metal) on top of the ALD-grown films. To keep the process simple, we grew these particles at a sample temperature of 100ºC, which is low for a PLD process, and an Ar pressure of 0.01 mbar in the chamber. 90 laser pulses were shot, in order to ensure that the Sr layer remains a set of loosely spaced Sr particles rather than forming large islands or a continuous film. In practice, the moment the samples exited the vacuum chamber, the Sr particles quickly reacted with the air in the room, probably becoming SrO, $Sr(OH)_2$, and eventually settling into $SrCO_3$. This was followed by annealing in a tube furnace and SEM/EBSD observation, in both cases as described in the main text.

As the SEM image in Figure S11a clearly demonstrates, individual crystal domains create a feature resembling a 2D Voronoi polyhedral tessellation formed by i) nucleation in randomly distributed points followed by ii) stellular 2D growth until impact with crystal growing in neighbor domains. Typical dimensions of these domains vary between 20 and 100 $\mu$m and their boundaries are rich in Sr, as EDS measurement confirmed (not shown in Figure S11). In Figure S11b we show a forward-scattered electron image map from the area studied by EBSD. This area has size of 46 x 99 $\mu m^2$ and contains 41181 mapping points arranged in hexagonal grid with a mutual distance of 0.36 $\mu$m. Pixels located close to domain boundaries show a bright contrast due to stronger electron scattering from Sr atoms. The orientation imaging microscopy map in Figure S11c is a [001] Inverse pole figure (IPF, color) combined with an Image quality (IQ) map (B&W). Pixels that return low quality EBSD signal, which was not possible to index properly, are not colored, and they represent approximately 34% of all mapping points. Figure S11e is an example of a high quality Kikuchi pattern that was possible to index properly using a monoclinic $SrSiO_3$ structure[9]. The IPF map clearly demonstrates a strong preferential direction of the indexed crystal phase presented in the observed domains. This is confirmed by the texture plots shown in Figure S11d. We also point out that the indexing is most successful near the center of these domains, with signal quality noticeably decreasing closer to the boundaries. The (001) crystal plane is parallel to the sample surface in all domains. One of few typical crystal orientations detected in this

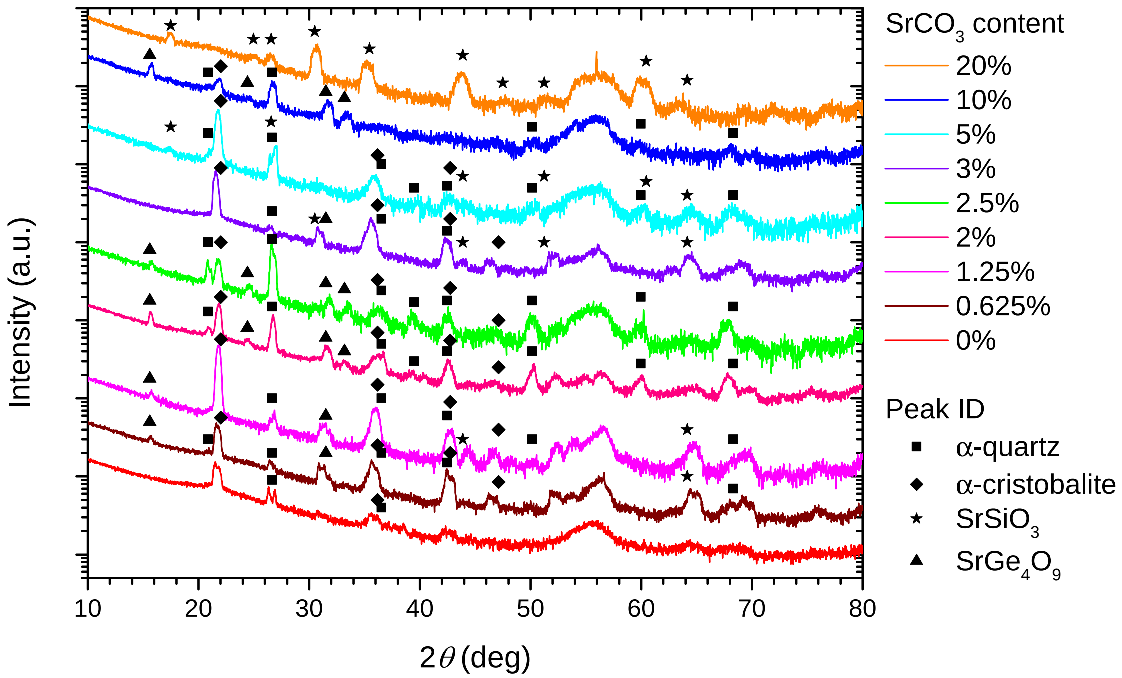


FIG. S9. Extended GIXRD scans.

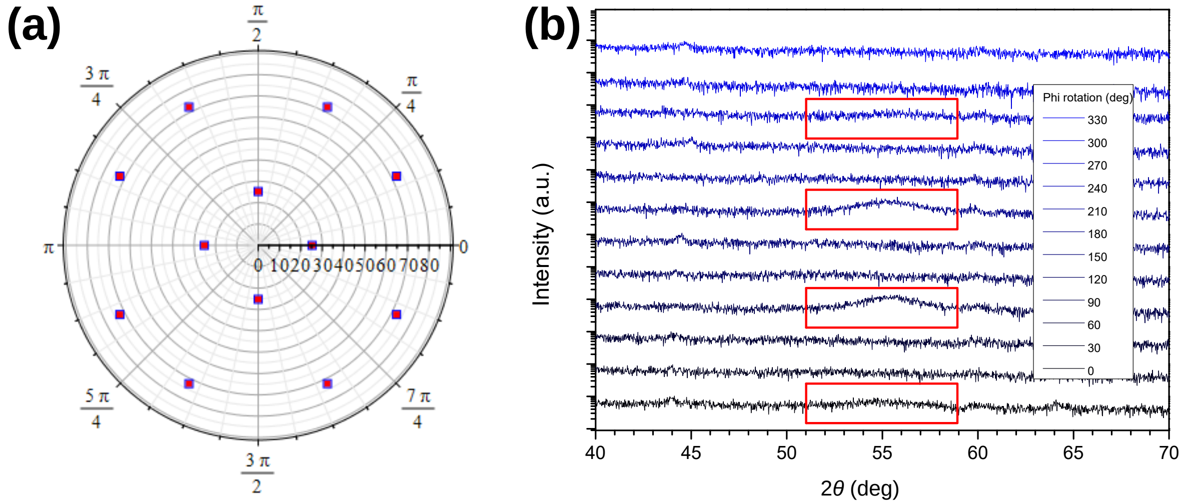


FIG. S10. a) Simulated pole figure for the (113) reflections of a Si(100) substrate. Note that this is a polar plot, with $\chi$ in the radial direction (equidistant ticks) and $\phi$ in the circumference, increasing counter-clockwise. Red squares indicate poles in the upper hemisphere, and blue boxes are for poles in the lower hemisphere, thus outside the measurement range. For Si(100), the projections overlap for the upper and lower hemispheres. Simulation details available at Reference 7. b) GIXRD scans taken on a sample on Si(100) at various $\phi$ rotation values. The sample was realigned prior to each scan. The red rectangles highlight the signal near 56°, which is present in the 0°, 90°, 180°, and 270° scans.

sample is shown in Figure S11f.

## REFERENCES:

[1] Q. Zhang, D. Sánchez-Fuentes, A. Gómez, R. Desgarceaux, B. Charlot, J. Gázquez, A. Carretero-Genevrier, and M. Gich, "Tailoring the crystal growth of quartz on silicon for patterning epitaxial piezoelectric films," Nanoscale Advances **1**, 3741–3752 (2019).

[2] H. Hosono and J. Nishii, "High photosensitivity and nanometer-scale phase separation in GeO2-SiO2 glass thin films," Optics Letters **24**, 1352–1354 (1999).

[3] M. Björk and G. Andersson, "GenX : an extensible X-ray reflectivity refinement program utilizing differential evolution," Journal of Applied Crystallography **40**, 1174–1178 (2007).

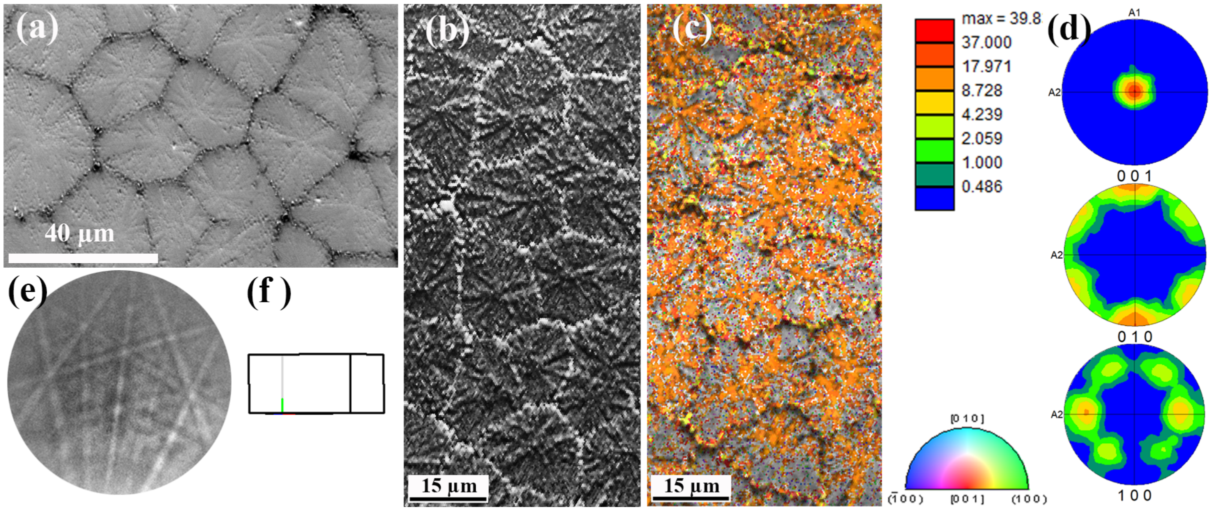


FIG. S11. Scanning electron microscopy characterization of crystal domains formed after crystallization of a $SiO_2/GeO_2$ ALD multilayer into $SrSiO_3$ (monoclinic[9]). a) Secondary electron image; b) Forward scattered electron image of area studied by EBSD; c) [001] IPF+IQ map showing the crystal direction parallel to the sample surface normal; d) (001), (010) and (100) sample texture plots using scale of multiples of random distribution (MRD); e) example of Kikuchi pattern and f) corresponding crystal orientation (axonometric projection in top view).

[4]G. Henderson, "The Germanate Anomaly: What do we know?" Journal of Non-Crystalline Solids **353**, 1695–1704 (2007).

[5]J. Z. Tischler, J. D. Budai, G. E. Ice, and A. Habenschuss, "Multiple scattering and the 200 reflection in silicon and germanium," Acta Crystallographica Section A **44**, 22–25 (1988).

[6]C. Weiss, *New Silicon Nanocrystal Materials for Photovoltaic Applications*, Ph.D. thesis, Friederich-Schiller-Universität Jena (Jena, Germany) (2017).

[7]P. Nukala, J. Antoja-Lleonart, Y. Wei, L. Yedra, B. Dkhil, and B. Noheda, "Direct Epitaxial Growth of Polar (1-x )HfO 2-( x )ZrO 2 Ultrathin Films on Silicon," ACS Applied Electronic Materials **1**, 2585–2593 (2019).

[8]J. Antoja-Lleonart, S. Zhou, K. de Hond, S. Huang, G. Koster, G. Rijnders, and B. Noheda, "Atomic layer deposition of SiO2-GeO2 multilayers," Applied Physics Letters **117**, 041601 (2020).

[9]F. Nishi, "Strontium Metasilicate, SrSiO3," Acta Crystallographica Section C Crystal Structure Communications **53**, 534–536 (1997).

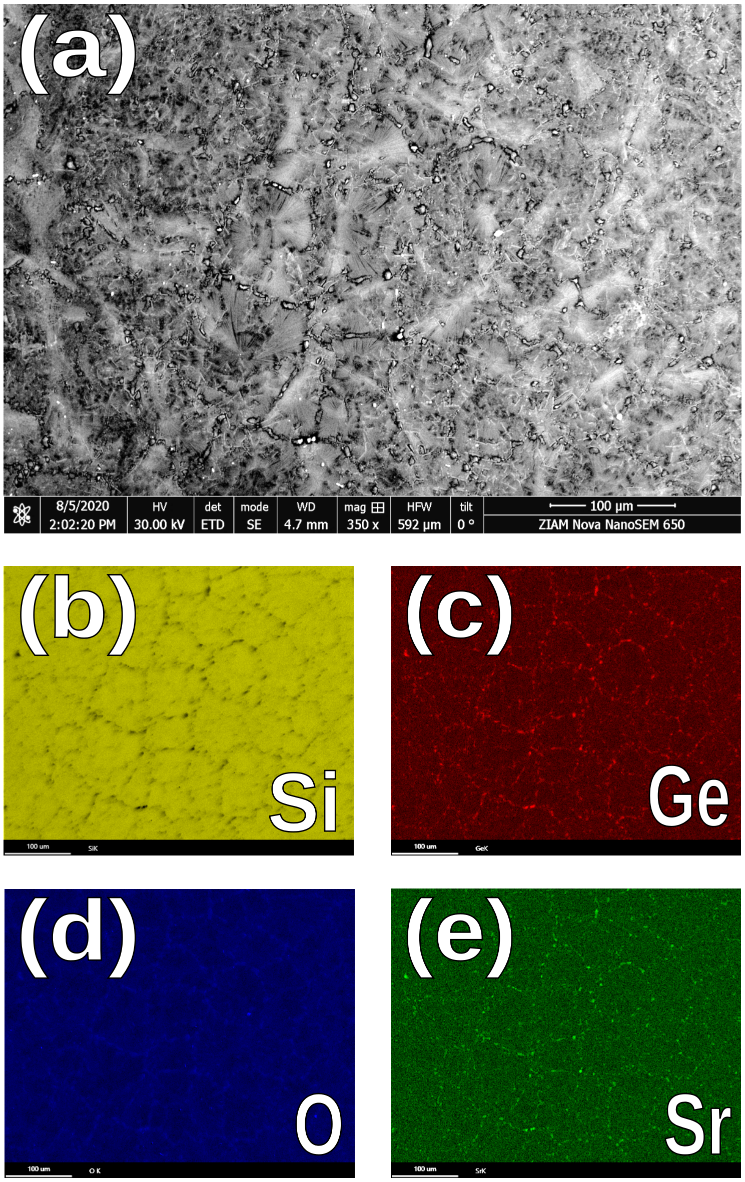


FIG. S12. a) SEM image of a x = 20% annealed film. Annealing has a clear effect on sample topography, which suggests the growth of spherulites whose crystallinity we were not able to ascertain. b-e) EDS composition maps for the main elements in the films. From these maps, it appears that the grain boundaries are Si-depleted, which can be attributed to increased sample thickness in those regions. EDS is sensitive to signal from the Si substrate in the case of relatively thin films, and likewise if a part of the film is thicker, EDS will display proportionally higher contributions from elements in the film (which includes Si, but at a much lower atomic percent than the substrate does).

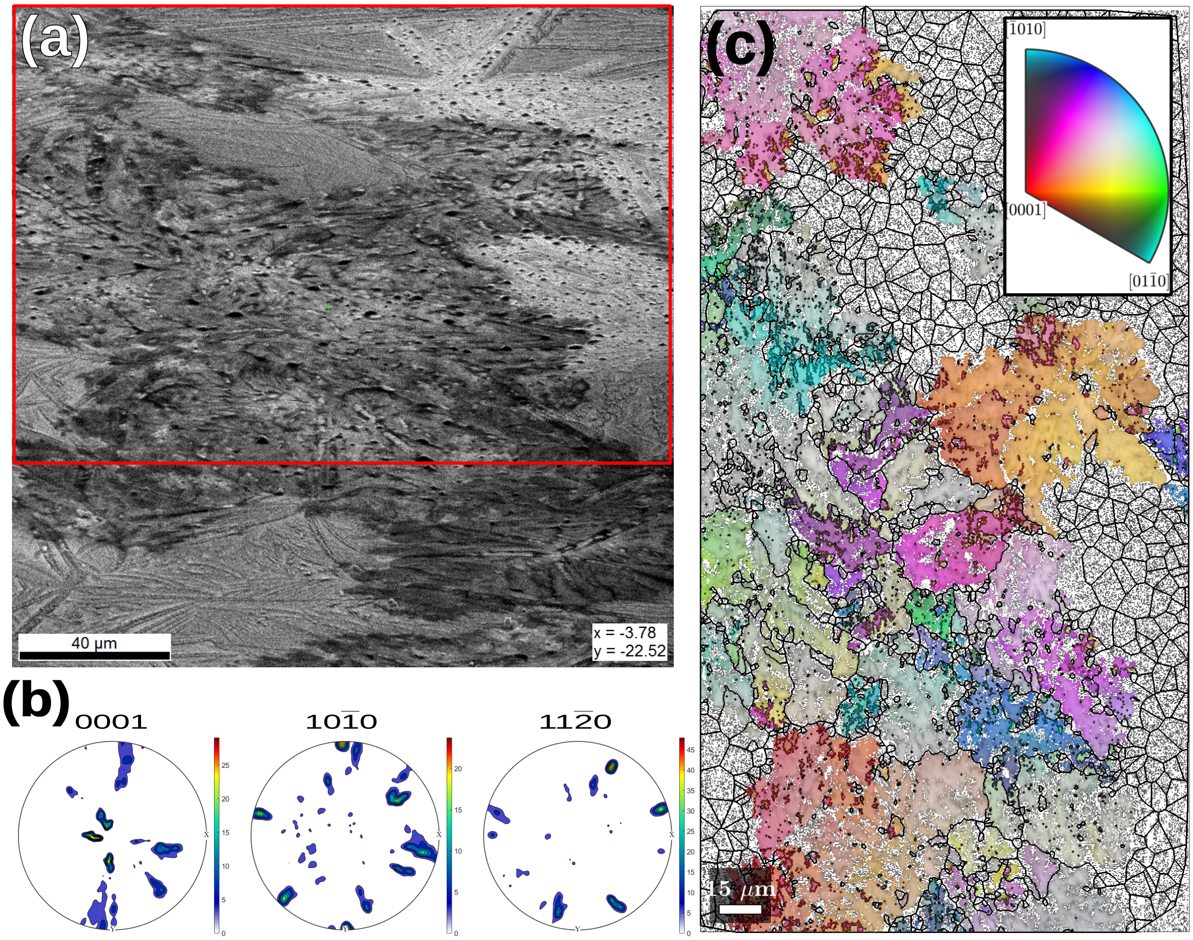


FIG. S13. EBSD analysis of the x = 10% sample of the series. Indexing is made with the $\alpha$-quartz unit cell. a) 71°-tilted SEM image of a region containing both crystalline needles and spherulites. The red rectangle shows the area analyzed with EBSD. b) ODF plotted in three different pole figures. The intensity scale is in multiples of random density (MRD). c) [001]- IPF (Inverse Pole Figure) of the region. There is no preferred orientation overall, but the patterns show that there are quartz regions in addition to the non-quartz, long needles, which we have been unable to index.

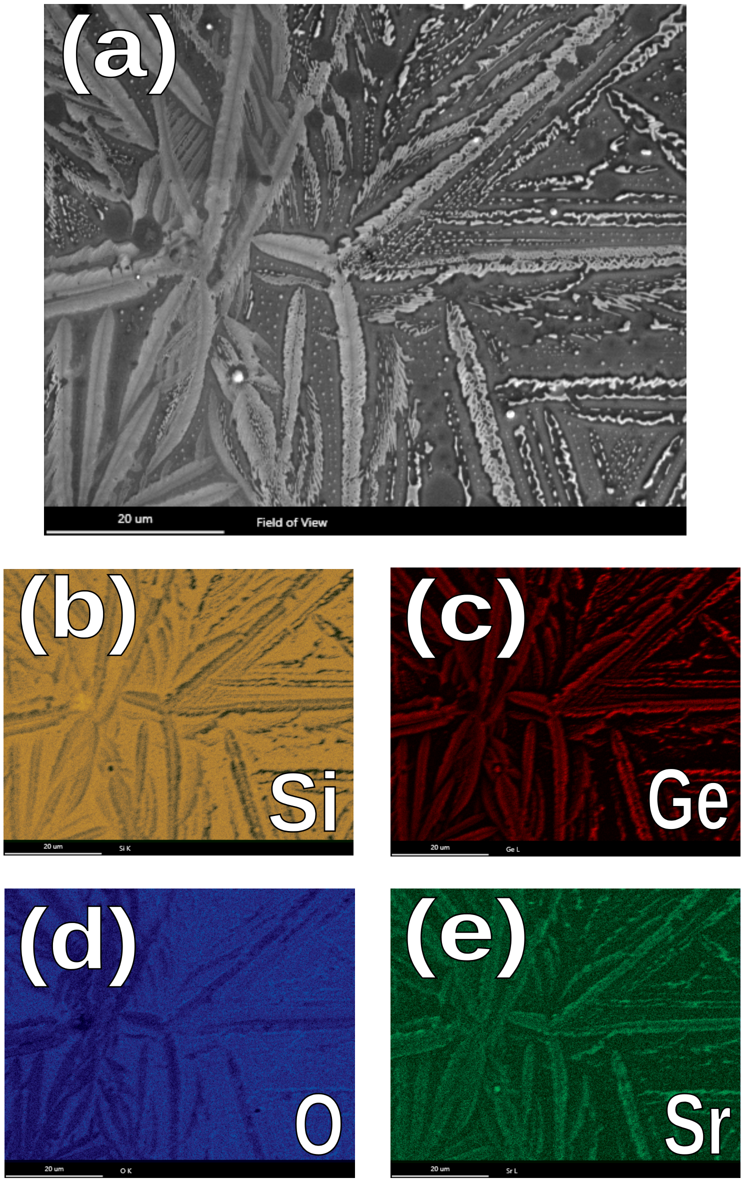


FIG. S14. a) SEM image of an x = 10% annealed film. After annealing, needles (in light grey) of length in the tens of micrometers become visible. In regions of this type, EBSD indicates that there are no crystalline $SiO_2$ phases, therefore the needles might correspond to a silicate or germanate phase. b-e) EDS composition maps for the main elements in the films. The needles appear to be not only silicon poor (which can be explained by their added thickness, see Figure S12), but also oxygen defficient.

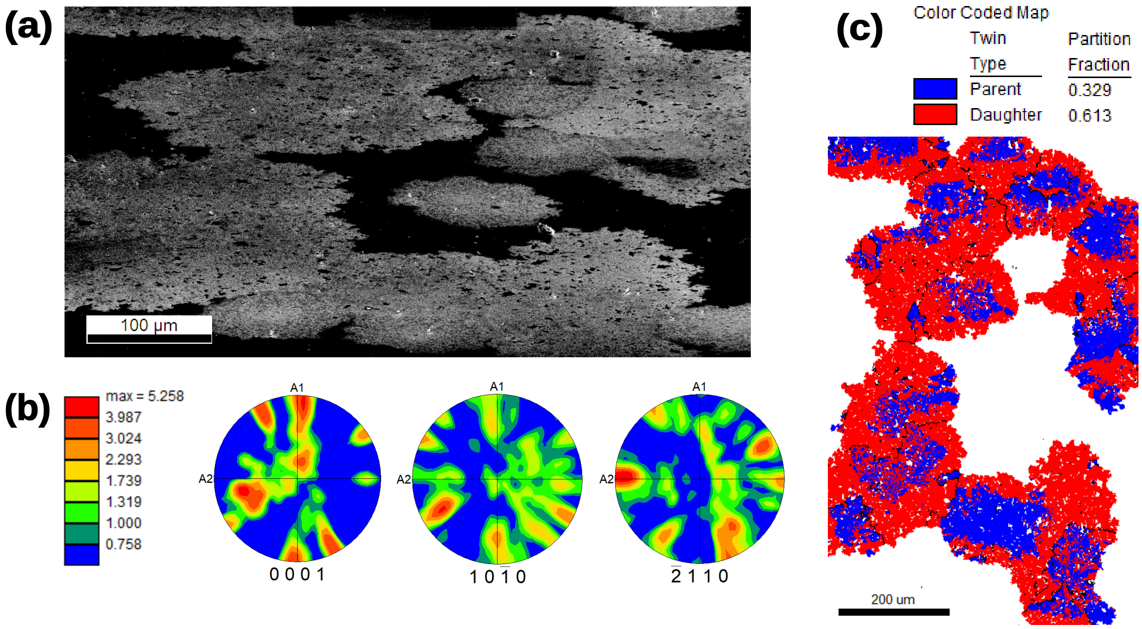


FIG. S15. EBSD analysis of the x = 2.5% sample of the series, including multiple coagulated islands. Indexing is made with the $\alpha$-quartz unit cell. We have not been able to index the region between the islands. a) 71°-tilted SEM image of a crystalline, non-dendritic region. b) ODF plotted in three different pole figures shows the presence of a few grains ($\sim$5) with different orientations. c) EBSD map of the region showing parent and daughter twinned areas. Grain boundaries indicated with black lines separate islands with different crystal orientations.
